# Interobserver reliability of echocardiography for prognostication of normotensive patients with pulmonary embolism

**DOI:** 10.1186/1476-7120-12-29

**Published:** 2014-08-04

**Authors:** Dita Kopecna, Sem Briongos, Hugo Castillo, Carlos Moreno, Mónica Recio, Paula Navas, José Luis Lobo, Angel Alonso-Gomez, Izaskun Obieta-Fresnedo, Covadonga Fernández-Golfin, José Luis Zamorano, David Jiménez

**Affiliations:** 1 Respiratory Department, Ramón y Cajal Hospital, IRYCIS, Alcalá de Henares University, 28034 Madrid, Spain; 2 Cardiology Department, Ramón y Cajal Hospital IRYCIS, Madrid, Spain; 3 Respiratory Department, Txagorritxu Hospital, Vitoria, Spain; 4 Cardiologyy Department, Txagorritxu Hospital, Vitoria, Spain

**Keywords:** Pulmonary embolism, Prognosis, Echocardiography, Interobserver reliability, Reproducibility

## Abstract

**Objectives:**

To evaluate the interobserver reliability of echocardiographic findings of right ventricle (**RV**) dysfunction for prognosticating normotensive patients with pulmonary embolism (**PE**).

**Methods:**

A central panel of cardiologists evaluated echocardiographic studies of 75 patients included in the PROTECT study for the following signs: RV diameter, RV/left ventricular (**LV**) diameter ratio, hypokinesis of the RV free wall, and tricuspid plane systolic excursion (**TAPSE**). Investigators used intraclass correlation to assess agreement between the measurements of the central panel and each of the local cardiologists. Investigators used the single weighted kappa statistic to test for agreement between readers of interpretation of RV enlargement and RV hypokinesis.

**Results:**

The two observers had fair agreement (k = 0.45) for RV enlargement assessed by the RV diameter, and good agreement (k = 0.65) for RV enlargement assessed by the RV/LV diameter ratio. The interobserver reliability of the assessment whether hypokinesis of the RV free wall is present was good (к = 0.70), and whether RV dysfunction (assessed by TAPSE measurement) is present was very good (k = 0.86). The intraclass correlation for the RV/LV diameter ratio was fair (0.55; 95% confidence interval [**CI**], 0.37-0.69), for the RV diameter was good (0.70; 95% CI, 0.56-0.80), and for the TAPSE measurement was very good (0.85; 95% CI, 0.77-0.90). On Bland-Altman analysis, the mean differences for RV diameter, RV/LV diameter ratio and TAPSE measurement were 2.33 (±5.38), 0.06 (±0.23) and 0.08 (±2.20), respectively.

**Conclusion:**

TAPSE measurement is the least user dependent and most reproducible echocardiographic finding of RV dysfunction in normotensive patients with PE.

## Introduction

Acute pulmonary embolism (**PE**) is a common disease with a 3-month mortality rate of up to 17.4% [1-4]. Even if PE is properly treated with anticoagulation, the mortality rate in hemodynamically stable patients varies from 8.1% to 15.1% [4,5]. Death is usually caused by acute right heart failure [4-9]. Acute PE increases the pressure of the pulmonary arterial system and right ventricle (**RV**) resulting in RV dysfunction, which may progress to right heart failure and circulatory collapse [5,6]. Patients with RV dysfunction have a higher mortality rate than those without, even if they are initially hemodynamically stable [6,7]. Thus, the presence of RV dysfunction is a marker for adverse clinical outcome in patients with acute PE [6-8].

Transthoracic echocardiography (**TTE**) is the most common first-line examination to diagnose the signs of RV dysfunction [6-9]. Echocardiography is capable of visualizing the changes occurring in the morphology and function of the right ventricle as a result of acute pressure overload. A variety of different methods for the assessment of RV dysfunction on TTE have been proposed and the literature shows variable results for the prognostic power of TTE signs of RV dysfunction to predict adverse outcomes [10]. This variability may in part be explained by the somewhat subjective nature of diagnosing RV dysfunction on TTE because formal criteria for establishing these signs are not available. It is noteworthy that prior publications on this topic did not report interobserver reproducibility of the findings.

Accordingly, the purpose of our study was to determine the interobserver reproducibility of TTE findings previously described to indicate RV dysfunction with the goal of identifying the most robust, least observer dependent method.

## Methods

### Study design

This was a sub analysis of the first 75 patients enrolled in the PROTECT, a prospective, multicenter observational cohort study designed by the authors (see Appendix) and sponsored by the Institute of Health Carlos III, Spain (NCT00880737) [11]. Local ethics committees approved the study. All patients provided written informed consent.

### Patients

Only patients diagnosed with pulmonary embolism by multidetector CT were eligible [12]. Exclusion criteria consisted of treatment with thrombolytics at the time of PE diagnosis, life expectancy less than 3 months, pregnancy, geographic inaccessibility precluding follow-up, age younger than 18 years, renal insufficiency (creatinine clearance < 30 mL/min), inability to complete CT testing (e.g., allergy to intravenous contrast agents, unavailability of CT, patient too ill), or hemodynamic instability at presentation (defined as cardiogenic shock, systolic blood pressure < 90 mmHg, or use of inotropic support). We also excluded patients that did not successfully complete the protocol-required transthoracic echocardiography.

### Examinations

The study required that patients undergo echocardiography (i.e., TTE) within 24 hours after diagnosis of PE. Patients underwent testing in the left lateral position. Trained and certified local cardiologists, blinded to the patient’s clinical data and laboratory test results, performed and interpreted each echocardiogram. This sub study defined echocardiographic RV dysfunction as the presence of dilatation of the right ventricle (end-diastolic diameter > 30 mm from the parasternal view or the right ventricle appearing larger than the left ventricle from the subcostal or apical view), hypokinesis of the right ventricle free wall (any view), or a tricuspid plane systolic excursion (**TAPSE**) of 1.6 cm or less. TAPSE was measured as the total displacement of the tricuspid annulus (centimeters) from end-diastole to end-systole, with values representing the average TAPSE of three to five beats [13].

Local cardiologists recorded all examinations on digital format for off-line blinded re-evaluation by one of the echocardiographers from the central panel (S.B., M.R. and H.C.) (25 studies each).

### Statistical analyses

This sub study assessed interobserver reproducibility by the intraclass correlation coefficient and Bland-Altman analysis [14]. For the presence or absence of RV dysfunction, the study assessed interobserver reproducibility using weighted kappa measurement. The kappa value for agreement was interpreted as follows: poor, < 0.20; fair, 0.21–0.40; moderate, 0.41–0.60; good, 0.61–0.80; and very good, 0.81–1.00 [15].

Statistical significance was defined as a two-tailed *P*-value of <0.05 for all analyses. Analyses were performed using SPSS, version 15.0 for the PC (SPSS, Inc. Chicago, IL, USA).

## Results

Transthoracic echocardiography was technically inadequate in 2 of the 75 patients who were enrolled in this sub study (2.7%; 95% confidence interval [**CI**], 0 to 6.3%). The median age was 70 years (interquartile range [**IR**], 59–79), and 47% of patients were female (Table 1). According to the local cardiologists’ measurements, the RV end-diastolic diameter was > 30 mm in 60% of patients (44/73). In 22% (16/73) of patients, the ratio of the RV to the LV short axis was greater than 1, and 11% of patients had a TAPSE of 1.6 cm or less.

**Table 1:** Baseline characteristics for normotensive patients with acute symptomatic pulmonary embolism

	***All patients N = 73***
**Clinical characteristics, n (%)**	
Age, years, median (25^th^-75^th^ percentiles)	70 (59–79)
Male gender	39 (53%)
**Risk factors for VTE, n (%)**	
Cancer^†^	9 (12%)
Recent surgery^‡^	4 (5.5%)
Immobilization^y^	25 (34%)
**Comorbid diseases, n (%)**	
COPD	14 (19%)
Congestive heart failure	3 (4.1%)
**Clinical symptoms and signs at presentation, n (%)**	
Syncope	16 (22%)
Chest pain	38 (52%)
Dyspnea	54 (74%)
Heart rate > 100/minute	20 (27%)
Arterial oxyhemoglobin saturation (SaO_2_) < 90%	16 (22%)
SBP < 120 mm Hg	18 (25%)
Concomitant DVT	35 (48%)

*Abbreviations: VTE* venous thromboembolism, *COPD* chronic obstructive pulmonary disease, *SBP* systolic blood pressure, *DVT* deep vein thrombosis.

^†^Active or under treatment in the last year.

^‡^In the previous month.

^y^Immobilized patients are defined in this analysis as non-surgical patients who had been immobilized (i.e., total bed rest with bathroom privileges) for ≥4 days in the month prior to PE diagnosis.

### RV end-diastolic diameter

**Figure 1:**
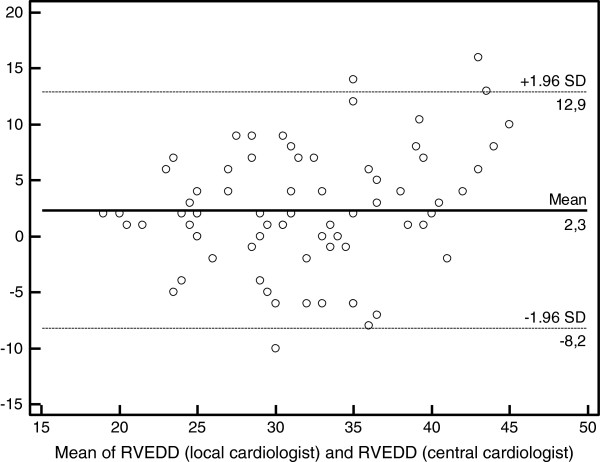
**Bland-Altman analysis of RV end-diastolic diameter measured by two cardiologists.** Abbreviations: RVEDD, right ventricle end-diastolic diameter; SD, standard deviation.

The mean RVD end-diastolic diameter from the parasternal view measured by local and central cardiologist were 3.3 ± 0.7 cm and 3.1 ± 0.6 cm, respectively. The intraclass correlation was good (0.70; 95% CI, 0.56-0.80). On Bland-Altman analysis of RV end-diastolic diameter measurements, the means and standard deviation (**SD**) between central and local cardiologists were 2.33 and 5.38, respectively (Figure 1). When RV dysfunction was defined as a RV end-diastolic diameter > 30 mm from the parasternal view, the two observers agreed that in 23 patients (32%; 95% CI, 21-42%) no RV dysfunction was present. The two observers agreed that RV dysfunction was present in 30 patients (41%; 95% CI, 30-52%); disagreement whether residual occurred in 20 patients (27%; 95% CI, 17-38%) (Table 2). The interobserver agreement whether RV dysfunction is present or not is fair, with a weighted kappa of 0.45 (95% CI, 0.25-0.65).

**Table 2:** Interobserver variability for the presence of RV dysfunction (defined as a RV end-diastolic diameter > 30 mm from the parasternal view)

**Central cardiologist**				
		**No RV dysfunction (n)**	**RV dysfunction (n)**	**Total (n)**
**Local cardiologist**	**No RV dysfunction (n)**	23	6	29
	**RV dysfunction (n)**	14	30	44
	**Total (n)**	37	36	73

*Abbreviations*: *RV* right ventricle.

### Ratio of the RV to the LV short axis

**Figure 2:**
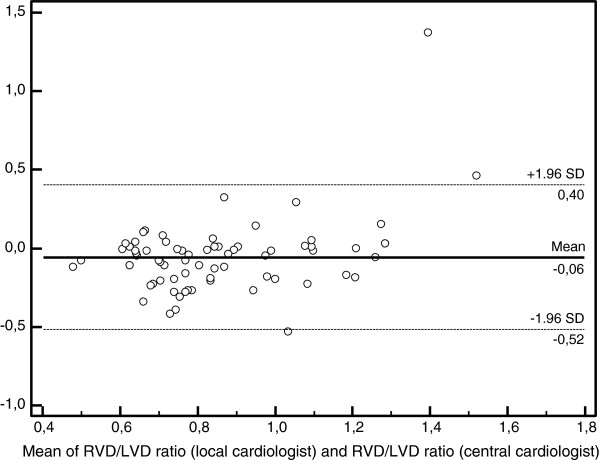
**Bland-Altman analysis of ratio of the RV to the LV short axis measured by two cardiologists.** Abbreviations: RVD, right ventricle diameter; LVD, left ventricle diameter; SD, standard deviation.

The mean RV to left ventricle ratios were 0.83 ± 0.28 for local and 0.88 ± 0.20 for central cardiologist, respectively. The intraclass correlation was fair (0.55; 95% CI, 0.37-0.69). On Bland-Altman analysis of RV/LV ratio measurements, the means and standard deviation (**SD**) between central and local cardiologists were 0.06 and 0.23, respectively (Figure 2). For the ratio of the RV to the LV short axis the observers agreed that 52 patients (71%; 95% CI, 61-82%) were free of RV dysfunction. They agreed upon the presence of RV dysfunction in 12 patients (16%; 95% CI, 7.9-25%). Disagreement existed in 9 patients (12%; 95% CI, 4.8-20%) (Table 3). The interobserver agreement reflecting the presence or absence of RV dysfunction was good with a weighted kappa of 0.65 (95% CI, 0.44-0.86).

**Table 3:** Interobserver variability for the presence of RV dysfunction (defined as a RV appearing larger than the left ventricle from the subcostal or apical view)

**Central cardiologist**				
		**No RV dysfunction (n)**	**RV dysfunction (n)**	**Total (n)**
**Local cardiologist**	**No RV dysfunction (n)**	52	5	57
	**RV dysfunction (n)**	4	12	16
	**Total (n)**	56	17	73

*Abbreviations*: *RV* right ventricle.

### Hypokinesis of the RV free wall

The observers agreed that 55 patients (75%; 95% CI; 65-85%) were free of hypokinesis of the RV free wall. They agreed upon the presence of RV free wall hypokinesis in 11 patients (15%; 95% CI, 6.9-23%). Disagreement existed in 7 patients (9.6%; 95% CI, 2.8-16%) (Table 4). The interobserver agreement reflecting the presence or absence of RV free wall hypokinesis was good with a weighted kappa of 0.70 (95% CI, 0.50 to 0.91).

**Table 4:** Interobserver variability for the presence of RV free wall hypokinesis

**Central cardiologist**				
		**No RV dysfunction (n)**	**RV dysfunction (n)**	**Total (n)**
**Local cardiologist**	**No RV dysfunction (n)**	55	4	59
	**RV dysfunction (n)**	3	11	14
	**Total (n)**	58	15	73

*Abbreviations:RV* right ventricle.

### TAPSE

**Figure 3:**
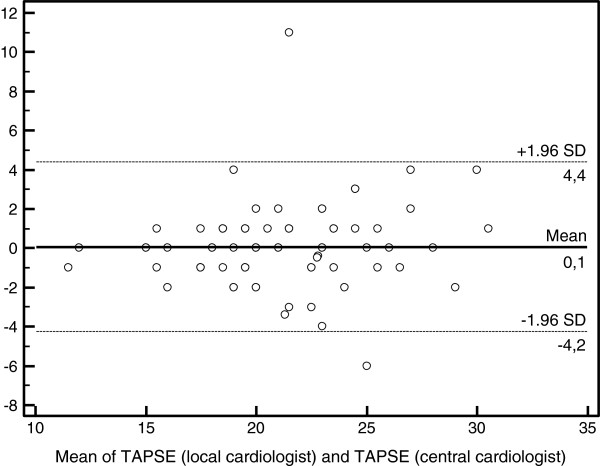
**Bland-Altman analysis of TAPSE measured by two cardiologists.** Abbreviations: TAPSE, tricuspid annular systolic excursion; SD, standard deviation.

The mean TAPSE measured by local and central cardiologist was 2.1 ± 0.4 cm and 2.1 ± 0.4 cm, respectively. The intraclass correlation for the TAPSE measurement was very good (0.85; 95% CI, 0.77-0.90). On Bland-Altman analysis of TAPSE measurements, the means and standard deviation (**SD**) between central and local cardiologists were 0.08 and 2.20, respectively (Figure 3). When RV dysfunction was defined as a TAPSE of 1.6 cm or less, the two observers agreed that in 64 patients (88%; 95% CI, 80-95%) no RV dysfunction was present. The two observers agreed that RV dysfunction was present in 7 patients (9.6%; 95% CI, 2.8-16%); disagreement whether residual occurred in 2 patients (2.7%; 95% CI, 0–6.5%) (Table 5). The interobserver agreement whether RV dysfunction is present or not is very good, with a weighted kappa of 0.86 (95% CI, 0.67 to 1).

**Table 5:** Interobserver variability for the presence of RV dysfunction (assessed by a TAPSE of 1.6 cm or less)

**Central cardiologist**				
		**No RV dysfunction (n)**	**RV dysfunction (n)**	**Total (n)**
**Local cardiologist**	**No RV dysfunction (n)**	64	1	65
	**RV dysfunction (n)**	1	7	8
	**Total (n)**	65	8	73

*Abbreviations*: *RV* right ventricle.

## Discussion

In this study, we aimed at analyzing the observer dependence of establishing the echocardiographic signs of RV dysfunction that have hitherto been described in the literature to identify the most robust and reproducible method. Our results suggest that considerable interindividual differences exist in the reproducibility of echocardiographic signs of RV dysfunction. TAPSE measurement is the least user dependent and most reproducible.

Studies have recognized RV dysfunction as a key determinant of prognosis in PE [16]. Echocardiographic findings suggesting RV dysfunction have been reported to occur in at least 25% of PE patients [17]. A meta-analysis found more than a two-fold increased risk of PE-related mortality in patients with echocardiographic signs of RV dysfunction [18]. Two out of the seven studies included an estimation of risk in normotensive patients with PE [7,19]. In such patients RV dysfunction had sensitivity of 56–61% and was related to the absolute increase in the early PE-related mortality of 4–5% [18]. Importantly, patients with normal echocardiographic findings had an excellent outcome, with in hospital PE-related mortality less than 1% in most of the reported series [6,7,19]. The most important limitations are the lack of standardization of the echocardiographic criteria [10], and the somewhat subjective nature of diagnosing RV dysfunction on pulmonary echocardiography.

Our results show that the interobserver reliability was higher for qualitative abnormalities on transthoracic echocardiography (i.e., hypokinesis of the RV free wall). There are several potential explanations for these findings. Moderate or severe RV dysfunction is usually identified qualitatively, and it is ordinarily readily apparent to observers even with only modest experience [8]. Moreover, RV dimensions are highly dependent on probe rotation by the user, which can result in an underestimation of RV width [20]. For normotensive patients with acute symptomatic PE, TAPSE is independently predictive of survival [21]; however no studies had previously assessed interobserver reliability for this parameter in these patients. In the initial validation study by Kaul et al. [22], TAPSE correlated strongly with radionuclide angiography, with low interobserver variability. For PE patients, our results confirm that interobserver variability for TAPSE measurements was very low and lower than for all other echocardiographic signs of RV dysfunction investigated here.

The findings of this study might have clinical implications. Some authors have proposed that normotensive patients with RV dysfunction on echocardiography should potentially undergo thrombolytic therapy [23]. This study detected only fair reproducibility of the RV end-diastolic diameter and the RV diameter/left ventricular diameter ratio. Thus, findings from this study do not adequately justify use of these parameters to drive decision-making regarding thrombolytic therapy.

Besides the fact that this study is the first study to report upon the clinical implication of interobserver reliability on the measurement of echocardiographic RV dysfunction in normotensive patients with acute PE, this study has other strengths. Particularly, this is the first study that reports on the interobserver reliability of TAPSE. Our study has several limitations. We were not able to assess the interobserver reproducibility of systolic pulmonary pressure and other echocardiographic criteria for RV dysfunction (e.g., systolic excursion velocity of the tricuspid annulus). Though with 3D echocardiography there is less underestimation of RV end-diastolic and end-systolic volumes and improved test-retest variability compared with 2D echocardiography [24], investigators did not perform volumetric analyses of the RV. Furthermore, our results should be interpreted with caution due to the limited sample size.

In conclusion, this is the first study that systematically assessed the interobserver reliability of echocardiographic findings of RV dysfunction in normotensive patients with acute PE. We found considerable differences in the interobserver reproducibility of these findings. TAPSE measurement is the least user dependent and most reproducible. If these signs are used in clinical practice to make patient management decisions, practitioners should be aware of the variable degree of subjectivity and reproducibility associated with these observations.

## Appendix

**Coordinator of the PROTECT Study:** David Jiménez

**PROTECT Steering Committee Members**: David Jiménez, José Luis Lobo, Manuel Monreal, Remedios Otero, Roger D. Yusen

**PROTECT Study Coordinating Center:** S & H Medical Science Service

**Adjudication Committee:** Francisco Conget, Dolores Nauffal, Mikel Oribe, Fernando Uresandi

**Radiological Panel**: Ignacio Gallego, Luis Gorospe, Agustina Vicente

**Blood Sample Processing**: José Manuel del Rey

**Statisticians**: Víctor Abraira, Javier Zamora, Alfonso Muriel

## Competing interests

The authors declare that they have no competing interests.

## Authors’ contributions

Study concept and design: DK, JLL, JLZ, DJ. Acquisition of data; analysis and interpretation of data; statistical analysis: DK, SB, HC, CM, MR, PN, JLL, AA-G, IO-F, CF-G, JLZ, DJ. Drafting of the manuscript: DK, SB, HC, CM, MR, PN, JLL, AA-G, IO-F, CF-G, JLZ, DJ. Critical revision of the manuscript for important intellectual content: DK, SBs, HC, CM, MR, PN, JLL, AA-G, IO-F, CF-G, JLZ, DJ. Study supervision: JLZ, DJ. The corresponding author, DJ, had full access to all the data in the study and had final responsibility for the decision to submit for publication. All authors read and approved the final manuscript.

## Authors’ information

**Investigators of the PROTECT study:** Consolación Rodríguez, Jorge Vivancos, Jesús Marín (Bormujos), Aitor Ballaz, Jose María Abaitúa, Sonia Velasco (Galdakao), Manuel Barrón, María Lladó, Carmen Rodrigo, Luis Javier Alonso (Logroño), Ramón Rabuñal, Olalla Castro, Concepción Iglesias, Ana Testa (Lugo), David Jiménez, Vicente Gómez, Luis Gorospe, Sem Briongos, José Manuel del Rey (Madrid), Celso Álvarez, Nuria Rodríguez, Amador Prieto, María Martín (Oviedo), Carmen Navarro, Mónica López, Eva Castañer, Eva Guillaumet (Sabadell), Remedios Otero, Teresa Elías, Pilar Serrano, Francisco López (Sevilla), Reina Valle, María Victoria Piret, Pilar Lucio, José María Cuesta (Sierrallana), Marta Ballester, José Pamies, Ana Osa (Valencia), José Luis Lobo, Vanesa Zorrilla, Delfina Del Pozo, Ángel Alonso (Vitoria), Miguel Ángel Santolaria, Mariano González, José Luis de Benito (Zaragoza).
